# Obstructive Sleep Apnea as a Predictor of Abnormal Heart Rate Turbulence

**DOI:** 10.3390/jcm9010001

**Published:** 2019-12-18

**Authors:** Dominika Urbanik, Paweł Gać, Helena Martynowicz, Małgorzata Poręba, Maciej Podgórski, Marta Negrusz-Kawecka, Grzegorz Mazur, Małgorzata Sobieszczańska, Rafał Poręba

**Affiliations:** 1 Department of Internal Medicine, Occupational Diseases, Hypertension and Clinical Oncology, Wroclaw Medical University, 50-556 Wroclaw, Poland; dominika.urbanik@umed.wroc.pl (D.U.); helena.martynowicz@umed.wroc.pl (H.M.); maciej.podgorski@umed.wroc.pl (M.P.); grzegorz.mazur@umed.wroc.pl (G.M.); rafal.poreba@umed.wroc.pl (R.P.); 2 Department of Hygiene, Wroclaw Medical University, 50-345 Wroclaw, Poland; 3 Department of Pathophysiology, Wroclaw Medical University, 50-368 Wroclaw, Poland; malgorzata.poreba@umed.wroc.pl; 4 Department of Cardiology, Wroclaw Medical University, 50-556 Wroclaw, Poland; marta.negruszkawecka@umed.wroc.pl; 5 Department of Geriatrics, Wroclaw Medical University, 50-369 Wroclaw, Poland; malgorzata.sobieszczanska@umed.wroc.pl

**Keywords:** obstructive sleep apnea, heart rate turbulence, apnea-hypopnea index

## Abstract

Obstructive sleep apnea (OSA) causes dysfunction of the autonomic nervous system, but the exact mechanism has not been fully understood. The aim of this study was to analyse the relationship between the incidence and severity of OSA and heart rate turbulence (HRT). Seventy one patients with clinical suspicion of OSA were qualified to participate in the study. All participants took part in a survey and were subjected to laboratory tests, 24-hour electrocardiogram (ECG) Holter monitoring with HRT analysis and polysomnography. The group with OSA manifested significantly higher turbulence onset (TO) and lower turbulence slope (TS) as compared to the group without OSA. Older age, diabetes, hypertension and higher apnea/hypopnea index (AHI) were found to be independent risk factors for increased TO, whereas older age, higher body mass index (BMI), higher blood glucose levels, hypertension and higher AHI were independent risk factors for TS reduction. The AHI ≥65 criterion indicates abnormal HRT in patients with OSA with 94.9% sensitivity and 50.0% specificity, which gives a prediction accuracy of 85.7%. In summary, OSA should be considered as a predictor of abnormal HRT.

## 1. Introduction

Obstructive sleep apnea (OSA) is a sleep apnea syndrome that increases cardiovascular risk. It is caused by repeated partial or complete closure of the upper respiratory tract, leading to hypoxaemia, hypercapnia, deterioration of sleep quality and sympathetic hyperactivity [1,2]. OSA is associated with a higher incidence of cardiovascular diseases, including hypertension [3], ischaemic heart disease [4], heart failure [5], stroke [6] and cardiac arrhythmias [7]. It has been proved that sleep apnea causes dysfunction of the autonomic nervous system, but the exact mechanism has not been fully understood [8].

Heart rate turbulence (HRT) is an important prognostic indicator for the activity of the autonomic system in cardiovascular diseases. This method was presented by Schmidt et al. in 1999 to assess cardiovascular risk in patients after myocardial infarction [9]. Heart rate turbulence is an analysis of the variability of the sinus node response by evaluating the frequency of discharges after the occurrence of ventricular premature beat (VPB) and is regulated by a baroreceptor reflex. VPBs are extra heartbeats that occur due to abnormal electrical activation that originates in the ventricles. HRT analyses assess how the sinus node, which triggers heartbeats and is controlled by the baroreceptor, responds to VPB by assessing the frequency of the following discharges. Heart rate turbulence is defined as turbulence onset (TO) and turbulence slope (TS) [10]. TO is percentage difference between the heart rate immediately following premature ventricular complex and the heart rate immediately preceding premature ventricular complex. TO is expressed as a percentage, which means that the heart rate acceleration after additional ventricular excitation is <0% under normal conditions. TS is the steepest slope of the linear regression line for each sequence of five consecutive normal intervals in the local tachogram. TS is expressed in milliseconds per RR interval (time elapsed between two successive R-waves of the QRS signal on the electrocardiogram) and its standard is >2.5 ms/RR [10]. The prognostic value of TO and TS indices of cardiac death was shown especially in the population of patients after myocardial infarction [9,11,12], patients with heart failure [13,14,15,16], cardiomyopathy [17] and in patients who required heart transplantation [18].

A review of previous studies shows that obstructive sleep apnea is an important risk factor for arrhythmia development. A study by Selim et al. showed that moderate and severe OSA is associated with a twofold higher risk of any heart rhythm disturbances during sleep [7]. Sleep apnea is conducive to complex ventricular arrhythmias [19], atrial fibrillation [20] and conduction abnormalities such as pauses, bradycardia and atrioventricular blocks [21]. In the 5-year follow-up of more than 10,000 patients diagnosed for sleep apnea, a significant correlation was found between decreased mean saturation at night and sudden cardiac death (SCD) [22].

Sleep apnea has been shown to disturb the balance of the autonomic nervous system (ANS), inducing sympathetic hyperreactivity and reduction of parasympathetic activity [23]. Turbulence of heart rhythm with proven predictive value in patients with ischemic heart disease and heart failure is a good parameter of ANS assessment [24]. There are still few studies analysing correlations between obstructive sleep apnea and heart rate turbulence [25,26,27]. 

The aim of this study was to analyse the relationship between the incidence and severity of OSA and heart rate turbulence in patients with clinical suspicion of OSA. In addition, the purpose of the study was to determine apnea/hypopnea index (AHI) predictive values for the occurrence of abnormal HRT.

## 2. Material and Methods

### 2.1. Study Group

We retrospectively qualified 71 consecutive patients referred to department of internal medicine with clinical suspicion of obstructive sleep apnea. The patients were enrolled between January 2017 and December 2018. Inclusion criteria were as follows: age between 18 and 90 years, the presence of at least 25 additional ventricular beats in Holter electrocardiogram (ECG), clinical suspicion of OSA, and patients willing to participate in this study. Exclusion criteria were as follows: presence of active inflammation, presence of active malignancy and severe mental disorders, and cognitive disability. The study group consisted of 43 men and 28 women. The average age was 58.86 ± 11.69 years. In the majority of subjects obesity (59.1%), hyperlipidaemia (67.6%) and hypertension (74.6%) were found. Diabetes mellitus occurred in 19.7% of patients and ischemic heart disease in 16.9% (Table 1).

**Figure 1:**
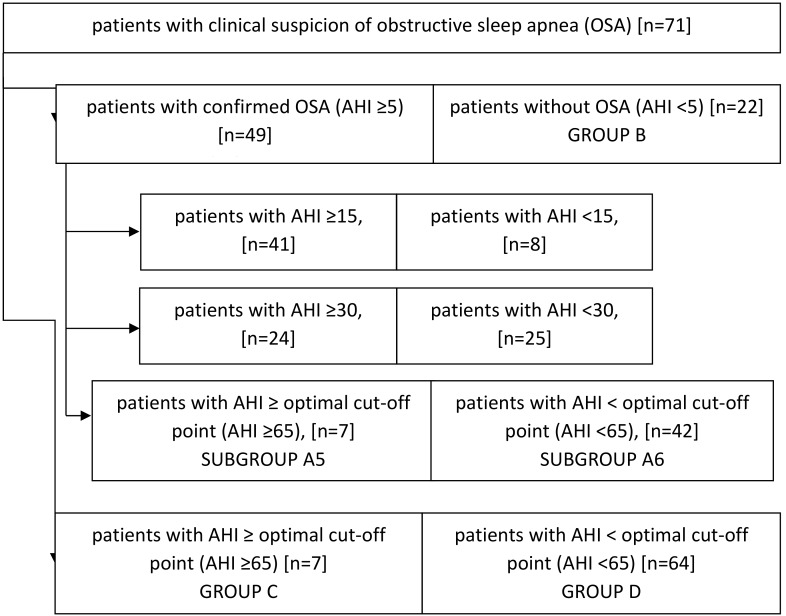
Study groups and differentiated study subgroups.

The group was divided into patients with the presence of OSA (group A) and without OSA (group B). The disease was defined on the basis of elevated AHI ≥ 5/h. Using the optimal cut-off point for AHI according to the receiver operating characteristic (ROC) curve, two further subgroups were identified from the whole study group, where patients with AHI ≥ optimal cut-off point were found in group C and patients with AHI < optimal cut-off point according to the ROC curve in group D. For statistical purposes, the group of patients with OSA was further disaggregated, taking into account three different AHI limits: 15, 30 and the optimal cut-off point according to the ROC curve. 6 subgroups were obtained: subgroup A1 with AHI ≥ 15/h, A2 with AHI < 15/h, A3 with AHI ≥ 30/h, A4 with AHI < 30/h, A5 with AHI ≥ optimal cut-off point, A6 with AHI < optimal cut-off point according to the ROC curve (Figure 1).

### 2.2. Study Methodology

The study was approved by the Local Ethics Committee.

The study group carried out surveys, laboratory tests, polysomnography and 24-hour Holter ECG monitoring at the same time. The survey asked about demographic and anthropometric parameters (gender, age, height, body weight), cardiovascular diseases (ischaemic heart disease, stroke) and cardiovascular risk factors (hypertension, diabetes, hyperlipidaemia, smoking).

The concentrations of total cholesterol, LDL cholesterol, HDL, triglycerides and fasting glucose were determined.

Patients had a full polysomnographic examination, unattended, type II with NoxA1 ResMed (serial number 992901595) in the Sleep Medicine Laboratory. Records of brain bioelectrical function (electroencephalography, EEG), eyeball movements (electrooculography, EEA), muscular tension from anterior chinothoracic and tibial electrodes (electromyogram, EMG), air flow from the nasal and nasal pressure sensor, chest and abdomen movements by inductive plethysmography, blood saturation by pulse oximetry were recorded. The analysis of the tests was carried out by a qualified physician, in accordance with the guidelines of the American Academy of Sleep Medicine on the Noxturnal system (version 5.1.2.2.20294) [25].

### 2.3. Heart Rate Turbulence (HRT)

Twenty-four hour (from 6:00 to 6:00 the next day) Holter ECG recordings were made with Lifecard CF 12-channel recorder serial number LIFE-045348/2015, and recording analysis with Sentinel Spacelabs Healthcare Pathfinder SL version 1.7.1.5164 with serial number 8395 (Delmar Reynolds, Hertford, UK). Holter monitoring and polysomnography were carried out in each patient simultaneously. The time of the polysomnographic recorder was synchronized with the time of the Holter ECG recorder. The study group was to observe the set hours of daytime activity (6:00–22:00) and night-time rest (from 22:00 to 6:00 next day) and to keep a diary of activities performed during the day and hours of sleep. The examinations were analysed by one doctor who had no insight into the patient’s clinical data and the result of polysomnography. The heart rate turbulence (HRT) have been automatically calculated by the Sentinel Spacelabs system. To properly prepare the ECG for subsequent analysis, the editing of automatic record was verified visually. HRT parameters were considered normal when TO <0%, and TS >2.5 ms/RR interval. The analysed HRT indices permitted to define percentages of individuals with both normal HRT parameters (HRT0), individuals with a single abnormal parameter of HRT (HRT1) and individuals with both abnormal HRT parameters (HRT2), (Table 2).

### 2.4. Statistical Analysis

Statistical analyses were carried out using the statistical package “Dell Statistica 13.1”. (Dell Inc., Texas, USA). The distribution of variables was checked by Lilliefors and W-Shapiro–Wilk tests. In the case of independent quantitative variables with normal distribution, the t-test for variables was used for further statistical analysis. In the case of variables with distribution other than normal, the Mann–Whitney U-test was used for quantitative independent variables. For independent qualitative variables, the quadrate-square test of the highest reliability was used for further statistical analysis. Correlation and regression analysis was performed to determine the relationships between the variables studied. Parameters of the model obtained in regression analysis were estimated using the least squares method. In addition, the accuracy of the test was evaluated based on ROC curve analysis. The results on the level of *p* < 0.05 were assumed to be statistically significant.

## 3. Results

In the whole study group of 71 patients admitted to hospital with suspected obstructive sleep apnea, the TO parameter was equal to –2.77 ± 2.31 % and the TS parameter was equal to 8.43 ± 6.47 ms/RR. Normal TO value was found in 66 patients, which constituted 92.9% of the study group, normal TS in 62 patients, 87.3% of the study group (Table 3).

Group A (with OSA) manifested significantly higher mean values of TO and lower mean values of TS as compared to group B (without OSA). As compared to group B, a significantly lower percentage of persons in group A was included in the normal TS category (Table 3).

**Figure 2:**
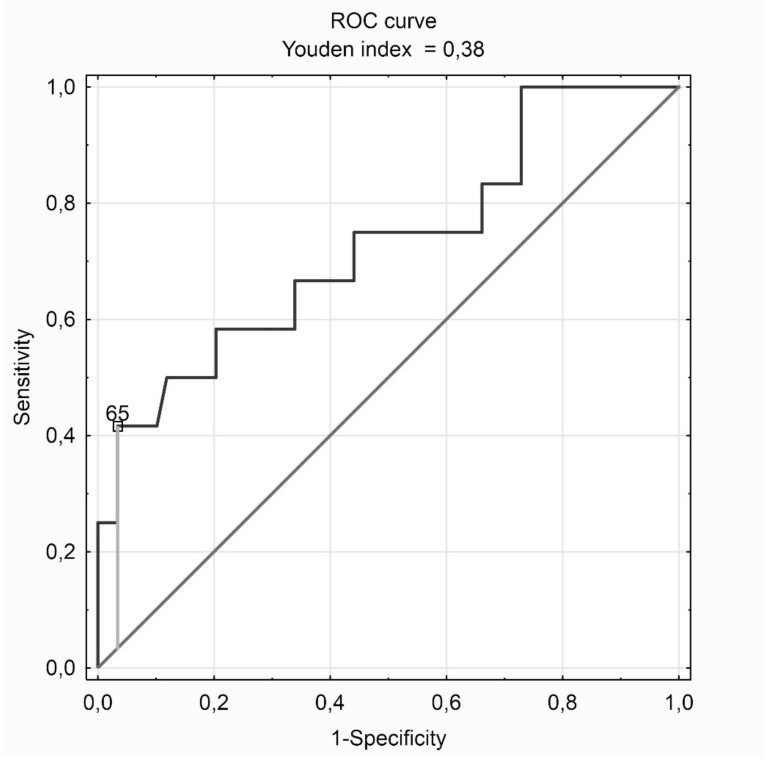
The receiver operating characteristic (ROC) curve of abnormal heart rate turbulence (HRT1/2—abnormal turbulence onset or/and abnormal turbulence slope) prediction among patients with suspected obstructive sleep apnea (clinical suspicion of OSA).

The ROC curve was plotted, which indicated AHI value equal to 65 as the optimal cut-off point for abnormal heart rate turbulence prediction in the studied group of patients with suspected OSA. The AHI ≥ 65 criterion indicates abnormal heart rate turbulence with a sensitivity of 96.6% and specificity of 41.7%, giving a prediction accuracy of 87.3%. For comparison, a typical OSA diagnosis criterion, i.e. AHI ≥ 5 indicates abnormal heart rate turbulence with sensitivity of 33.9% and specificity of 83.3%, which gives a prediction accuracy of 42.3%. The ROC curve of abnormal heart rate turbulence prediction among the examined patients is presented in Figure 2, and measures of the conducted relationship analysis in Table 4.

As compared to the remaining groups distinguished on grounds of the optimal cut-off point for abnormal heart rate turbulence prediction in the studied group of patients with suspected OSA, group C (with AHI ≥ 65) manifested significantly lower mean values of TS as compared to group D (with AHI < 65). As compared to group D, a significantly lower percentage of persons in group C was included to the normal TS category. A significantly lower proportion of persons were included in category HRT0 in group C than in group D. On the other hand, a significantly higher proportion of persons was included into the category of HRT1 in group C than in group D (Table 5).

The next stage of studies in group with OSA confirmed by polysomnography permitted us to distinguish the subgroups. Subgroup A1 (with moderate and severe obstructive sleep apnea, AHI ≥ 15) manifested significantly higher mean values of TO and lower mean values of TS as compared to subgroup A2 (with mild obstructive sleep apnea, AHI < 15), (Table 6A). A significantly lower proportion of persons were included to category normal TS in subgroup A3 (with severe obstructive sleep apnea, AHI ≥ 30) than in subgroup A4 (with mild and moderate obstructive sleep apnea, AHI < 30), (Table 6B).

**Figure 3:**
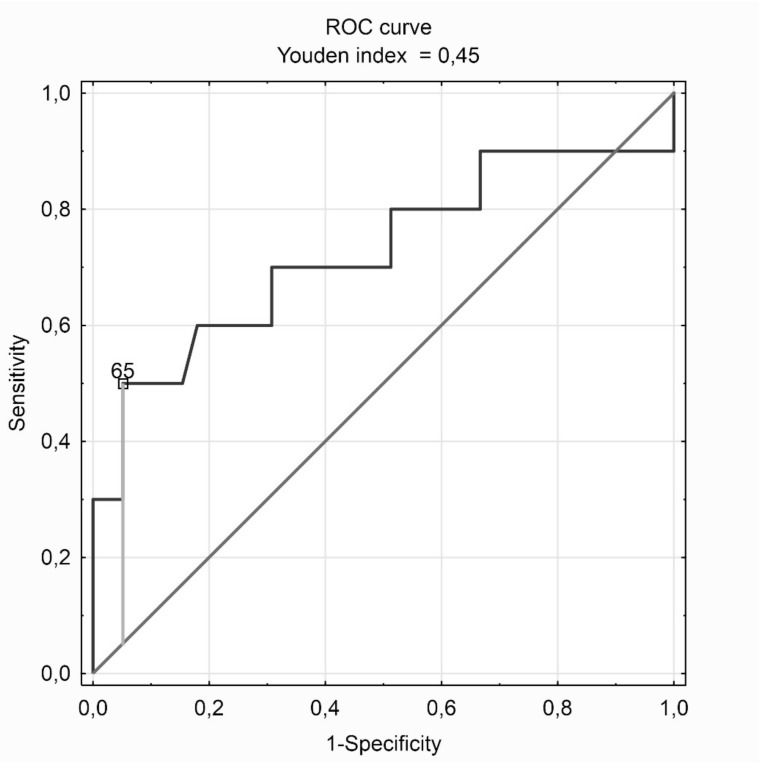
The ROC curve of abnormal heart rate turbulence (HRT1/2—abnormal turbulence onset or/and abnormal turbulence slope) prediction among patients with confirmed obstructive sleep apnea (AHI ≥ 5).

In the study group of patients with OSA confirmed by polysomnography, the ROC curve was plotted and the AHI value was equal to 65 as the optimal cut-off point for abnormal heart rate turbulence prediction. The AHI ≥65 criterion indicates abnormal heart rate turbulence in patients with OSA with 94.9% sensitivity and 50.0% specificity, which gives a prediction accuracy of 85.7%. For comparison, the typical criteria differentiating gravity of OSA, i.e. AHI ≥15 and AHI ≥30 indicate abnormal heart rate turbulence with sensitivity, specificity and accuracy, respectively 17.9%, 90.0%, 32.7% and 56.4%, 70.0%, 59.2%. The ROC curve of abnormal heart rate turbulence prediction among patients with confirmed OSA is presented in Figure 3, and measures of the relationship analysis carried out are in Table 7.

As compared to the remaining groups distinguished on the grounds of the optimal cut-off point for abnormal heart rate turbulence prediction in the studied group of patients with OSA confirmed by polysomnography, subgroup A5 (OSA with AHI ≥ 65) manifested significantly lower mean values of TS as compared to subgroup A6 (OSA with AHI < 65). As compared to subgroup A6, a significantly lower percentage of persons in subgroup A5 was included to the normal TS category. A significantly lower proportion of persons were included in category HRT0 in subgroup A5 than in subgroup A6. On the other hand, a significantly higher proportion of persons was included into the category of HRT1 in subgroup A5 than in subgroup A6 (Table 8).

The analysis of correlation between AHI and HRT parameters showed that there is a moderate positive correlation in the TO parameter (*r* = 0.33, *p* = 0.005) and a moderate negative correlation in the TS parameter (*r* = −0.42, *p* = 0.000) in the whole study group. In the group of patients with diagnosed obstructive sleep apnea there was a weak positive correlation in the TO parameter (*r* = 0.28, *p* = 0.045) and a moderate negative correlation in the TS parameter (*r* = −0.32, *p* = 0.023) (Table 9).

In the study group the possible independent risk factors for the parameters of heart rate turbulence were found on the basis of the univariable linear regressions between parameters of heart rate turbulence (TO, TS) and basic anthropometric data (age, height, body mass, BMI, gender, obesity), lipid metabolism parameters (blood concentration of total cholesterol, LDL cholesterol, HDL cholesterol, triglycerides), blood glucose concentration, cardiovascular diseases (hyperlipidaemia, diabetes, arterial hypertension, coronary artery diseases, stroke), smoking, AHI from polysomnography. As the next step, with the use of multivariable stepwise regression analysis taking into account statistically significant variables of univariable linear regressions, the final models were obtained for the specific parameters of heart rate turbulence. The following model have been obtained: 

TO = 0.090 age + 1.486 diabetes + 0.343 arterial hypertension + 0.019 AHI,

TS = −0.158 age − 0.073 BMI − 0.028 glucose − 3.480 arterial hypertension − 0.044 AHI

Older age, diabetes, hypertension and higher AHI values were found to be independent risk factors for increased TO, whereas older age, higher BMI, higher blood glucose levels, hypertension and higher AHI values were independent risk factors for TS reduction. The full results of the regression analysis are presented in Table 10A,B.

## 4. Discussion

The study showed that obstructive sleep apnea is an independent risk factor for abnormal heart rate turbulence. As AHI increases, the TO increases, while TS decreases. The severity of apnea affects the degree of disorders of the autonomic nervous system of the heart [23]. The AHI limit value, characterized by the highest prediction accuracy of abnormal 24-hour heart rate turbulence, was ≥65/h. Multivariable regression analysis showed other risk factors, apart from AHI, of abnormal HRT, including: older age, higher BMI values, higher blood glucose levels and the presence of diabetes and hypertension.

In healthy people with a properly functioning autonomic nervous system, after the appearance of additional ventricular stimulation, dynamic changes in heart rate in the form of initial acceleration and late gradual deceleration are observed [26]. If there is an autonomic nervous system (ANS) disorder, the characteristic response of the sinus node is weakened or eliminated [27]. Previous studies have shown that patients with sleep apnea develop abnormal regulation of the autonomic cardiovascular system. Yang et al. observed a negative correlation between night-time TS and AHI measurement in patients without obvious cardiovascular disease, with moderate and severe apnea with AHI > 20/h [28]. 

In our study we found statistically significant differences between the group with apnea (AHI ≥ 5) and apnea (AHI < 5) for both TS (*p* = 0.002) and TO (*p* = 0.025). Similar results were obtained by Aytemir et al., showing the effect of sleep apnea on the deterioration of turbulence indexes (HRT), heart rate variability (HRV) and QT interval dynamics, and thus on myocardial vulnerability on the occurrence of ventricular arrhythmias [29].

HRT values may be affected by many coexisting diseases: coronary heart disease [30], heart failure [31], hypertension [32], type 2 diabetes [33] and older age [34]. In our study, we demonstrated additional risk factors for abnormal turbulence parameters in the form of higher BMI values and abnormal fasting glycemia. Heart rate turbulence shows changes in sinus rhythm, which is a reflection of baroreceptors’ response to haemodynamic changes that occur after additional ventricular stimulation. Numerous experimental and clinical studies have shown that lowering the sensitivity of baroreceptors affects abnormal turbulence indices [35,36]. Among many markers of ANS function, researchers Yang and Szymanowska demonstrated that TS is the most sensitive indicator of autonomic nervous system damage in obstructive sleep apnea, its value exceeding the variability of heart rhythm [28,37]. Studies from recent years showed a statistically significant correlation between high AHI and reduced heart rate turbulence [31,38,39,40]. However, none of these studies determined the cut-off point for AHI, characterized by the highest sensitivity and specificity in the detection of HRT pathology. In this study, we have shown that AHI ≥65 has the highest prediction accuracy for abnormal heart rate turbulence. This is an important tip for clinicians to implement early cardiological prophylaxis in patients with severe sleep apnea and thus reduce their very high cardiovascular risk.

HRT might be related not only to pathological respiratory pattern in OSA, but also many others factors may be considered. Firstly, arousal and awaking may influence HRT. Secondly, oxygen desaturation and hypoventilation may be relevant. It is worth to notice that other than AHI OSA-related variables (time spent with oxygen saturation <90% sleep time, awakenings, periodic leg movements heart rate and daytime sleepiness) were significant independent predictors of composite cardiovascular outcome [41].

The main limitation of this study is a relatively small study group. Typically for patients with obstructive sleep apnea, the majority of patients had diagnosed arterial hypertension, obesity and hyperlipidaemia. These diseases may affect the autonomic nervous system, but at the same time the correlation between these states is so strong that it was not possible to select a group of patients without cardiovascular risk factors. On the other hand, the results were subjected to multifactorial regression analysis, which allowed us to determine independent predictive factors of reduced heart rate turbulence. In addition, the study group with co-morbidities reflects the true picture of the general population of patients with OSA and not a selected cohort. As a result, the results of our study add more value to everyday clinical practice.

## 5. Conclusions

- OSA should be considered as a predictor of abnormal heart rate turbulence.
- AHI ≥65 is characterized by the highest accuracy of abnormal heart rate turbulence in both the whole study group and the subgroup of patients with confirmed OSA.
- Higher AHI values, together with older age, higher BMI values, higher blood glucose levels, diabetes and hypertension are an independent risk factor for abnormal heart rate turbulence parameters and patients with suspected OSA.

## Figures and Tables

**Table 1:** Clinical characteristics of the study group.

	Whole Study Group	Obstructive Sleep Apnea (OSA) (Group A)	Without OSA (Group B)	*p* A vs. B
number	71 / 100.0	49 / 100.0	22 / 100.0	–
men	43 / 60.6	31 / 63.3	12 / 54.5	0.486
women	28 / 39.4	18 / 36.7	10 / 45.5	0.486
age (years)	58.86 ± 11.69	61.16 ± 10.43	53.73 ± 12.92	**0.012**
height (m)	1.71 ± 0.09	1.70 ± 0.09	1.74 ± 0.10	0.133
body mass (kg)	91.97 ± 18.16	96.80 ± 17.74	79.89 ± 13.08	**0.001**
BMI (kg/m^2^)	31.57 ± 6.57	33.60 ± 6.22	26.38 ± 4.23	**0.000**
obesity	42 / 59.1	36 / 73.5	6 / 27.3	**0.000**
total cholesterol (mg/dL)	214.02 ± 46.63	223.06 ± 51.41	192.71 ± 22.18	**0.040**
LDL cholesterol (mg/dL)	129.23 ± 40.63	138,24 ± 43.69	108.00 ± 21.27	**0.018**
HDL cholesterol (mg/dL)	52.45 ± 12.80	50.70 ± 11.40	56.57 ± 15.28	0.152
triglicerides (mg/dL)	165.94 ± 61.08	172.76 ± 64.23	149.86 ± 51.47	0.244
hyperlipidemia	48 / 67.6	36 / 73.5	12 / 54.5	0.115
glucose (mg/dL)	116.86 ± 33.11	120.81 ± 37.40	106.71 ± 14.57	0.179
diabetes mellitus	14 / 19.7	12 / 24.5	2 / 9.1	0.131
arterial hypertension	53 / 74.6	43 / 87.8	10 / 45.5	**0.006**
coronary artery diseases	12 / 16.9	4 / 8.2	8 / 36.4	0.093
stroke	1 / 1.4	1 / 2.0	0 / 0.0	0.978
smoking	9 / 12.7	7 / 14.3	2 / 9.1	0.984
AHI	24.79 ± 24.79	35.12 ± 23.32	1.80 ± 1.41	**0.000**

X/X—number/percentage; X ± X—mean ± standard deviation. Boldly marked with statistically significant differences (*p* < 0.05), BMI—body mass index, LDL—low-density lipoproteins, HDL—high-density lipoproteins, AHI—apnea/hypopnea index.

**Table 2:** Definitions and abbreviations of studied parameters of the heart rate turbulence.

Parameter	Unit	Definitions
TO	%	Turbulence onset: percentage difference between the heart rate immediately following premature ventricular complex and the heart rate immediately preceding premature ventricular complex. normal TO < 0 %.
TS	ms/RR	Turbulence slope: the steepest slope of the linear regression line for each sequence of five consecutive normal intervals in the local tachogram. normal TS > 2.5 ms per RR interval.
HRT0	–	Normal heart rate turbulence: normal TO and normal TS or inability to calculate TO and TS.
HRT1/2	–	Abnormal heart rate turbulence: abnormal TO or/and abnormal TS
HRT1	–	Abnormal TO or abnormal TS.
HRT2	–	Abnormal TO and abnormal TS.

HRT—heart rate turbulence, TO—turbulence onset, TS—turbulence slope.

**Table 3:** Parameters of heart rate turbulence in groups of patients breakdown by OSA (OSA defined as AHI ≥ 5).

	Whole Study Group	OSA (Group A) *n* = 49	Without OSA (Group B) *n* = 22	*p* A vs. B
TO (%)	−2.77 ± 2.31	−2.36 ± 2.27	−3.69 ± 2.20	**0.025**
normal TO	66 / 92.9	46 / 93.9	20 / 90.9	0.651
TS (ms/RR)	8.43 ± 6.47	6.85 ± 5.78	11.95 ± 6.66	**0.002**
normal TS	62 / 87.3	40 / 81.6	22 / 100.0	**0.031**
HRT0	59 / 83.1	39 / 79.6	20 / 90.9	0.239
HRT1/2	12 / 16.9	10 / 20.4	2 / 9.1	0.239
HRT1	10 / 14.1	8 / 16.3	2 / 9.1	0.428
HRT2	2 / 2.8	2 / 4.1	0 / 0.0	0.428

X/X—number/percentage; X ± X—mean ± standard deviation. Boldly marked with statistically significant differences (*p* < 0.05). AHI—apnea/hypopnea index, OSA—obstructive sleep apnea, TO—turbulence onset, TS—turbulence slope, HRT—heart rate turbulence.

**Table 4:** Sensitivity and specificity of AHI as abnormal heart rate turbulence prediction factor in the whole studied group of patients.

	For AHI ≥ 65	For AHI ≥ 5
Sensitivity	0.966	0.339
Specificity	0.417	0.833
Accuracy	0.873	0.423
Positive predictive values	0.891	0.909
Negative predictive values	0.714	0.204
Likelihood ratios positive	1.656	2.034
Likelihood ratios negative	0.081	0.793

AHI—apnea/hypopnea index.

**Table 5:** Parameters of heart rate turbulence in groups of patients breakdown by AHI = 65 (optimal cut-off point according to the ROC curve).

	AHI ≥ 65 (Group C) *n* = 7	AHI < 65 (Group D) *n* = 64	*p* C vs. D
TO (%)	−1.26 ± 0.85	−2.94 ± 2.36	0.068
normal TO	7 / 100.0	59 / 92.2	0.443
TS (ms/RR)	2.88 ± 1.98	9.04 ± 6.51	**0.016**
normal TS	2 / 28.6	60 / 93.7	**0.000**
HRT0	2 / 28.6	57 / 89.1	**0.000**
HRT1/2	5 / 71.4	7 / 10.9	**0.000**
HRT1	5 / 71.4	5 / 7.8	**0.000**
HRT2	0 / 0.0	2 / 3.1	0.135

X/X—number/percentage; X ± X—mean ± standard deviation. Boldly marked with statistically significant differences (*p* < 0.05). AHI—apnea/hypopnea index, TO—turbulence onset, TS— turbulence slope, HRT—heart rate turbulence.

**Table:** A. breakdown by AHI = 15

	OSA with AHI ≥15 (Subgroup A1) *n* = 41	OSA with AHI <15 (Subgroup A2) *n* = 8	*p* A1 vs. A2
TO (%)	−2.06 ± 1.72	−3.94 ± 3.87	**0.030**
normal TO	38 / 92.7	8 / 100.0	0.430
TS (ms/RR)	6.15 ± 3.72	10.43 ± 11.52	**0.049**
normal TS	33 / 80.5	7 / 87.5	0.639
HRT0	32 / 78.1	7 / 87.5	0.544
HRT1/2	9 / 21.9	1 / 12.5	0.544
HRT1	7 / 17.0	1 / 12.5	0.759
HRT2	2 / 4.9	2 / 0.0	0.759

**Table:** B. breakdown by AHI = 30

	OSA with AHI ≥30 (Subgroup A3) *n* = 24	OSA with AHI <30 (Subgroup A4) *n* = 25	p A3 vs. A4
TO (%)	−2.05 ± 1.89	−2.67 ± 2.58	0.347
normal TO	22 / 91.7	24 / 96.0	0.527
TS (ms/RR)	5.77 ± 4.08	7.88 ± 6.97	0.204
normal TS	17 / 70.8	23 / 92.0	**0.049**
HRT0	17 / 70.8	22 / 88.0	0.136
HRT1/2	7 / 29.1	3 / 12.0	0.136
HRT1	5 / 20.8	3 / 12.0	0.209
HRT2	2 / 8.3	0 / 0.0	0.209

X/X—number/percentage; X ± X—mean ± standard deviation. Boldly marked with statistically significant differences (*p* < 0.05). AHI—apnea/hypopnea index, OSA—obstructive sleep apnea, TO—turbulence onset, TS—turbulence slope, HRT—heart rate turbulence.

**Table 7:** Sensitivity and specificity of AHI as abnormal heart rate turbulence prediction factor in the group of patients with OSA.

	for AHI ≥ 65	for AHI ≥ 15	for AHI ≥ 30
Sensitivity	0.949	0.179	0.564
Specificity	0.500	0.900	0.700
Accuracy	0.857	0.327	0.592
Positive predictive values	0.881	0.875	0.880
Negative predictive values	0.714	0.220	0.292
Likelihood ratios positive	1.897	1.795	1.880
Likelihood ratios negative	0.103	0.912	0.623

AHI—apnea/hypopnea index.

**Table 8:** Parameters of heart rate turbulence in subgroups of OSA patients breakdown by AHI = 65 (optimal cut-off point according to the ROC curve).

	OSA with AHI ≥ 65 (Subgroup A5) *n* = 7	OSA with AHI < 65 (Subgroup A6) *n* = 42	*p* A5 vs. A6
**TO (%)**	−1.26 ± 0.85	−2.55 ± 2.38	0.167
**normal TO**	7 / 100.0	39 / 92.9	0.465
**TS (ms/RR)**	2.88 ± 1.98	7.51 ± 5.95	**0.048**
**normal TS**	2 / 28.6	38 / 90.5	**0.000**
**HRT0**	2 / 28.6	37 / 88.1	**0.000**
**HRT1/2**	5 / 71.4	5 / 11.9	**0.000**
**HRT1**	5 / 71.4	3 / 7.1	**0.000**
**HRT2**	0 / 0.0	2 / 4.8	0.068

X/X—number/percentage; X ± X—mean ± standard deviation. Boldly marked with statistically significant differences (*p* < 0.05). AHI—apnea/hypopnea index, OSA—obstructive sleep apnea, TO—turbulence onset, TS—turbulence slope, HRT—heart rate turbulence.

**Table 9:** Results of the analysis of correlation between apnea -hyponea index (AHI) and heart rate turbulence (HRT) parameters.

		AHI	
		*R*	*p*
whole study group	TO (%)	0.33	**0.005**
	TS (ms/RR)	−0.42	**0.000**
group with OSA	TO (%)	0.28	**0.045**
	TS (ms/RR)	−0.32	**0.023**
group without OSA	TO (%)	0.22	0.310
	TS (ms/RR)	−0.35	0.109

Boldly marked with statistically significant correlations (*p*< 0.05), AHI—apnea/hypopnea index, OSA—obstructive sleep apnea, TO—turbulence onset, TS—turbulence slope, HRT—heart rate turbulence.

**Table:** A. Model estimation for the dependent variable TO (%)

	Model for: TO (%)					
	Univariate Regression			Multivariable Stepwise Regression		
	Rc	SEM of Rc	p	Rc	SEM of Rc	p
men	−0.451	1.161	0.425	−	−	−
women	0.451	1.161	0.425	−	−	−
age (years)	0.091	0.029	**0.000**	0.090	0.027	**0.002**
height (m)	−3.955	4.435	0.197	−	−	−
body mass (kg)	0.027	0.030	0.072	−	−	−
BMI (kg/m^2^)	0.105	0.086	**0.012**	−0.073	0.012	**0.039**
obesity	1.183	0.368	**0.033**	−	−	−
total cholesterol (mg/dL)	−0.004	0.015	0.568	−	−	−
LDL cholesterol (mg/dL)	−0.003	0.016	0.739	−	−	−
HDL cholesterol (mg/dL)	0.006	0.031	0.980	−	−	−
triglicerides (mg/dL)	−0.004	0.007	0.409	−	−	−
hyperlipidemia	0.972	0.631	0.098	−	−	−
glucose (mg/dL)	0.009	0.010	0.349	−	−	−
diabetes mellitus	1.493	0.554	**0.029**	1.486	0.646	**0.025**
arterial hypertension	2.527	0.741	**0.000**	0.343	0.190	**0.046**
coronary artery diseases	0.194	0.792	0.793	−	−	−
stroke	0.887	2.145	0.706	−	−	−
smoking	0.148	0.857	0.876	−	−	−
AHI	0.031	0.004	**0.005**	0.019	0.008	**0.011**

**Table:** B. Model estimation for the dependent variable TS (ms/RR)

	Model for: TS (ms/RR)					
	Univariate Regression			Multivariable Stepwise Regression		
	Rc	SEM of Rc	*p*	Rc	SEM of Rc	*p*
men	0.003	3.005	0.977	−	−	−
women	−0.003	3.005	0.977	−	−	−
age (years)	−0.456	0.075	**0.000**	−0.158	0.093	**0.048**
height (m)	0.146	1.469	0.253	−	−	−
body mas (kg)	−0.358	0.075	**0.004**	−	−	−
BMI (kg/m^2^)	−0.428	0.224	**0.000**	−	−	−
obesity	−0.464	0.172	**0.000**	−	−	−
total cholesterol (mg/dL)	−0.080	0.038	0.592	−	−	−
LDL cholesterol (mg/dL)	−0.052	0.040	0.727	−	−	−
HDL cholestero (mg/dL)	0.107	0.082	0.475	−	−	−
triglicerides (mg/dL)	−0.257	0.017	0.081	−	−	−
hyperlipidemia	−0.295	0.063	**0.013**	−	−	−
glucose (mg/dL)	−0.324	0.027	**0.022**	−0.028	0.007	**0.027**
diabetes mellitus	− 0.210	0.195	0.079	−	−	−
arterial hypertension	−0.593	0.177	**0.000**	−3.480	0.975	**0.024**
coronary artery diseases	− 0.144	0.204	0.233	−	−	−
stroke	− 0.125	0.554	0.297	−	−	−
smoking	− 0.021	0.042	0.918	−	−	−
AHI	−0.423	0.036	**0.000**	−0.044	0.009	**0.024**

AHI—apnea/hypopnea index, BMI—body mass index, HDL—high density lipoproteins, LDL—low density lipoproteins, TO—turbulence onset, TS—turbulence slope.

## References

[1] Sateia M.J. (2014). International classification of sleep disorders-third edition: Highlights and modifications. Chest.

[2] Peppard P.E., Young T., Barnet J.H., Palta M., Hagen E.W., Hla K.M. (2013). Increased prevalence of sleep-disordered breathing in adults. Am. J. Epidemiol..

[3] Nieto F.J., Young T.B., Lind B.K., Shahar E., Samet J.M., Redline S., D’Agostino R.B., Newman A.B., Lebowitz M.D., Pickering T.G. (2000). Association of sleep-disordered breathing, sleep apnea, and hypertension in a large community-based study. Sleep Heart Health Study. JAMA.

[4] Sorajja D., Gami A.S., Sommers V.K., Behrenbeck T.R., Garcia-Touchard A., Lopez-Jimenez F. (2008). Independent association between obstructive sleep apnea and subclinical coronary artery disease. Chest.

[5] Gilat H., Vinker S., Buda I., Soudry E., Shani M., Bachar G. (2014). Obstructive sleep apnea and cardiovascular comorbidities a large epidemiologic study. Medicine (Baltimore).

[6] Arzt M., Young T., Finn L., Skatrud J.B., Bradley T.D. (2005). Association of sleep-disordered breathing and the occurrence of stroke. Am. J. Respir. Crit. Care Med..

[7] Selim B.J., Koo B.B., Qin L., Jeon S., Won C., Redeker N.S., Lampert R.J., Concato J.P., Bravata D.M., Ferguson J. (2016). The association between nocturnal cardiac arrhythmias and sleep-disordered breathing: The DREAM study. J. Clin. Sleep Med..

[8] Parish J.M., Somers V.K. (2004). Obstructive sleep apnea and cardiovascular disease. Mayo Clin. Proc..

[9] Schmidt G., Malik M., Barthel P., Schneider R., Ulm K., Rolnitzky L., Camm A.J., Bigger J.T., Schömig A. (1999). Heart rate turbulence after ventricular premature beats as a predictor of mortality after acute myocardial infarction. Lancet.

[10] Bauer A., Malik M., Schmidt G., Barthel P., Bonnemeier H., Cygankiewicz I., Guzik P., Lombardi F., Müller A., Oto A. (2008). Heart rate turbulence: Standards of measurement, physiological interpretation, and clinical use. International Society for Holter; Noninvasive Electrophysiology consensus. J. Am. Coll. Cardiol..

[11] La Rovere M.T., Bigger J.T., Marcus F.I., Mortara A., Schwartz P.J. (1998). Baroreflex sensitivity and heart-rate variability in prediction of total cardiac mortality after myocardial infarction. ATRAMI (Autonomic Tone and Reflexes After Myocardial Infarction) Investigators. Lancet.

[12] Ghuran A., Reid F., La Rovere M.T., Schmidt G., Bigger J.T., Camm A.J., Schwartz P.J., Malik M., Atrami Investigators (2002). Heart rate turbulence-based predictors of fatal and nonfatal cardiac arrest (The Autonomic Tone and Reflexes After Myocardial Infarction substudy). Am. J. Cardiol..

[13] Moore R.K., Groves D.G., Barlow P.E., Fox K.A., Shah A., Nolan J., Kearney M.T. (2006). Heart rate turbulence and death due to cardiac decompensation in patients with chronic heart failure. Eur. J. Heart Fail..

[14] Cygankiewicz I., Zareba W., Vazquez R., Vallverdu M., Gonzalez-Juanatey J.R., Valdes M., Almendral J., Cinca J., Caminal P., De Luna A.B. (2008). Heart rate turbulence predicts all-cause mortality and sudden death in congestive heart failure patients. Heart Rhythm.

[15] Grünefeld G.C., Kuck K.H., Ptaszynski P., Israel C.W., Connolly S.J., Roberts R.S., Dorian P., Hohnloser S.H. (2005). Refined risk stratification by heart rate turbulence in patients with reduced left ventricular function early after myocardial infarction: Results of the DINAMIT Holter substudy. Heart Rhythm.

[16] Disertori M., Mase M., Rigoni M., Nollo G., Ravelli F. (2016). Heart rate turbulence is a powerful predictor of cardiac death and ventricular arrhythmias in postmyocardial infarction and heart failure patients a systematic review and meta-analysis. Circ. Arrhythmia Electrophysiol..

[17] Klingenheben T., Ptaszynski P., Hohnloser S.H. (2008). Heart rate turbulence and other autonomic risk markers for arrhythmia risk stratification in dilated cardiomyopathy. J. Electrocardiol..

[18] Grimm W., Schmidt G., Maisch B., Sharkova J., Müller H.H., Christ M. (2003). Prognostic significance of heart rate turbulence following ventricular premature beats in patients with idiopathic dilated cardiomyopathy. J. Cardiovasc. Electrophysiol..

[19] Mehra R., Benjamin E.J., Shahar E., Gottlieb D.J., Nawabit R., Kirchner H.L., Sahadevan J., Redline S. (2006). Association of nocturnal arrhythmias with sleep-disordered breathing: The Sleep Heart Health Study. Am. J. Respir. Crit. Care Med..

[20] Gami A.S., Hodge D.O., Herges R.M., Olson E.J., Nykodym J., Kara T., Somers V.K. (2007). Obstructive sleep apnea, obesity, and the risk of incident atrial fibrillation. J. Am. Coll. Cardiol..

[21] Becker H.F., Koehler U., Stammnitz A., Peter J.H. (1998). Heart block in patients with sleep apnoea. Thorax.

[22] Gami A.S., Olson E.J., Shen W.K., Wright R.S., Ballman K.V., Hodge D.O., Herges R.M., Howard D.E., Somers V.K. (2013). Obstructive sleep apnea and the risk of sudden cardiac death: A longitudinal study of 10,701 adults. J. Am. Coll. Cardiol..

[23] Bisogni V., Pengo M.F., Maiolino G., Rossi G.P. (2016). The sympathetic nervous system and catecholamines metabolism in obstructive sleep apnoea. J. Thorac. Dis..

[24] Yin D.C., Wang Z.J., Guo S., Xie H.Y., Sun L., Feng W., Qiu W., Qu X.F. (2014). Prognostic significance of heart rate turbulence parameters in patients with chronic heart failure. BMC Cardiovasc. Disord..

[25] Yang A., Schäfer H., Manka R., Andrié R., Schwab J.O., Lewalter T., LüDERITZ B., Tasci S. (2005). Influence of obstructive sleep apnea on heart rate turbulence. Basic Res. Cardiol..

[26] Aytemir K., Deniz A., Yavuz B., Demir A.U., Sahiner L., Ciftci O., Tokgozoglu L., Can I., Sahin A., Oto A. (2007). Increased myocardial vulnerability and autonomic nervous system imbalance in obstructive sleep apnea syndrome. Respir. Med..

[27] Zuern C.S., Barthel P., Bauer A. (2011). Heart rate turbulence as risk-predictor after myocardial infarction. Front. Physiol..

[28] De Ponti R., Marazzato J., Bagliani G., Leonelli F.M., Padeletti L. (2018). Sick Sinus Syndrome. Card. Electrophysiol. Clin..

[29] Yoshihisa A., Suzuki S., Takiguchi M., Shimizu T., Abe S., Sato T., Yamaki T., Sugimoto K., Kunii H., Nakazato K. (2014). Impact of sleep-disordered breathing on heart rate turbulence in heart failure patients. PLoS ONE.

[30] Yu Y., Xu Y., Zhang M., Wang Y., Zou W., Gu Y. (2018). Value of assessing autonomic nervous function by heart rate variability and heart rate turbulence in hypertensive patients. Int. J. Hypertens.

[31] Balcioğlu S., Arslan U., Türkoğlu S., Ozdemir M., Cengel A. (2007). Heart rate variability and heart rate turbulence in patients with type 2 diabetes mellitus with versus without cardiac autonomic neuropathy. Am. J. Cardiol..

[32] Schwab J.O., Eichner G., Shlevkov N., Schrickel J., Yang A., Balta O., Lewalter T., Lüderitz B. (2005). Impact of age and basic heart rate on heart rate turbulence in healthy persons. Pacing Clin. Electrophysiol..

[33] Mrowka R., Persson P.B., Theres H., Patzak A. (2000). Blunted arterial baroreflex causes “physiological” heart rate turbulence. Am. J. Physiol. Regul. Integr. Comp. Physiol..

[34] Davies L., Francis D., Ponikowski P., Piepoli M., Coats A. (2001). Relation of heart rate and blood pressure turbulence following premature ventricular complexes to baroreflex sensitivity in chronic congestive heart failure. Am. J. Cardiol..

[35] Szymanowska K., Piatkowska A., Nowicka A., Cofta S., Wierzchowiecki M. (2008). Heart rate turbulence in patients with obstructive sleep apnea syndrome. Cardiol. J..

[36] Erdem A., Dogan O.T., Yontar O.C., Epozturk K., Ozlu M.F., Ozturk S., Ayhan S.S., Erdem F.H., Yazici M., Akkurt I. (2013). The pure effects of obstructive sleep apnea syndrome on cardiac autonomic functions: Heart rate turbulence analysis. Eur. Rev. Med. Pharmacol. Sci..

[37] Yates B.J., Bolton P.S., Macefield V.G. (2014). Vestibulo-sympathetic responses. Compr. Physiol..

[38] Berry R.B., Brooks R., Gamaldo C.E., Harding S.M., Lloyd R.M., Marcus C.L., Vaughn B.V. (2015). The AASM manual for the scoring of sleep and associated events: Rules, terminology, and technical specifications, version 2.2. Am. Acad. Sleep Med..

[39] Watanabe M.A., Bhalodia R., Lundequam E.J., Domitrovich P.P., Steinmeyer B.C., Stein P.K., Freedland K.E., Duntley S.P., Carney R.M. (2008). Increased ventricular premature contraction frequency during REM sleep in patients with coronary artery disease and obstructive sleep apnea. Indian Pacing Electrophysiol. J..

[40] Ozkececi G., Ulasli S.S., Akci O., Avsar A., Unlu M., Onrat E. (2016). The effect of sleep apnea severity on cardiac autonomic activity during night time in obstructive sleep apnea patients. Sao Paulo Med. J..

[41] Kendzerska T., Gershon A.S., Hawker G., Leung R.S., Tomlinson G. (2014). Obstructive sleep apnea and risk of cardiovascular events and all-cause mortality: A decade-long historical cohort study. PLoS Med..

